# Impact of gross tumor morphology on the clinical outcomes of colon cancer: multicenter retrospective cohort study

**DOI:** 10.1007/s00384-026-05101-1

**Published:** 2026-02-04

**Authors:** So Jung Han, Hyun Seok Lee, Byung Ik Jang, Jae Hyun Kim, Hyun Gun Kim, Il Hyun Baek, Jun Lee, Bun Kim, Dae Bum Kim, Jae Jun Park

**Affiliations:** 1 Department of Internal Medicine, Severance Hospital, Institute of Gastroenterology, Yonsei University College of Medicine, 50-1 Yonsei-Ro, Seodaemun-Gu, Seoul, 03722 South Korea; 2 Department of Internal Medicine, School of Medicine, Kyungpook National University, Kyungpook National University Hospital, 807 Hoguk-Ro, Buk-Gu, Daegu, 41404 Republic of Korea; 3 Department of Internal Medicine, Yeungnam University College of Medicine, Daegu, Republic of Korea; 4 Department of Internal Medicine, Kosin University College of Medicine, Busan, Republic of Korea; 5 Institute for Digestive Research, Soonchunhyang University College of Medicine, Seoul, Republic of Korea; 6 Department of Internal Medicine, Inha University College of Medicine, Incheon, Korea; 7 Department of Internal Medicine, Chosun University, Gwangju, Republic of Korea; 8 Center for Colorectal Cancer and Department of Internal Medicine, Research Institute and Hospital, National Cancer Center, Goyang, Republic of Korea; 9 Department of Internal Medicine, St. Vincent’s Hospital, College of Medicine, The Catholic University of Korea, Suwon, Korea

**Keywords:** Colon Cancer, Gross Morphology, Endoscopy, Clinical outcome, Prognostic Factor, Stage II

## Abstract

**Purpose:**

While histopathological features are established prognostic factors in colorectal cancer, the prognostic significance of gross tumor morphology remains unclear. We investigated whether endoscopic gross morphology is associated with clinical outcomes in colon cancer.

**Methods:**

We performed a multicenter retrospective analysis of 1,177 patients with colon cancer who underwent curative-intent endoscopic or surgical resection between 2010 and 2019. Tumors were categorized based on endoscopic images as flat/ulceroinfiltrative (*n* = 345) or fungating/ulcerofungating (*n* = 832). Kaplan–Meier analysis assessed survival outcomes, and Cox proportional hazards models identified independent prognostic factors, adjusting for age, sex, family history, diabetes, CEA, and AJCC 7th edition stage.

**Results:**

Patients with flat/ulceroinfiltrative tumors had significantly shorter overall survival (OS, *p* = 0.001) and disease-free survival (DFS, *p* = 0.024) than those with fungating/ulcerofungating tumors. In stage II patients, the difference in OS by morphology was more pronounced (*p* = 0.004). Multivariate analysis confirmed flat/ulceroinfiltrative morphology as an independent predictor of poor OS (HR 1.61; 95% CI 1.122–2.335; *p* = 0.010). Other significant predictors included older age (≥ 65 years, HR 1.533; *p* = 0.021), poor histologic grade (PD vs. WD/MD, HR 5.308; *p* < 0.001), and advanced stage.

**Conclusions:**

Gross endoscopic morphology is an independent prognostic factor in colon cancer. Flat/ulceroinfiltrative tumors are associated with worse outcomes, especially in stage II disease. Gross morphology, readily identifiable at diagnosis, may aid risk stratification and inform decisions regarding adjuvant therapy.

**Supplementary information:**

The online version contains supplementary material available at 10.1007/s00384-026-05101-1.

## Introduction

Colorectal cancer (CRC) remains a major global health challenge, ranking as the third most commonly diagnosed cancer and the second leading cause of cancer-related mortality worldwide [1]. In recent decades, significant advancements in surgical techniques, chemotherapy, and radiation therapy have improved patient outcomes. Prognostic stratification and treatment planning in colon cancer primarily rely on the American Joint Committee on Cancer (AJCC) TNM staging system, which classifies disease based on tumor invasion depth (T), lymph node involvement (N), and the presence of distant metastases (M) [2].

Beyond TNM staging, various histopathological features are recognized as important prognostic indicators. Histological grade, lymphovascular invasion, perineural invasion, and tumor budding are well-established predictors of aggressive tumor behavior and are increasingly used to guide clinical decisions, particularly regarding adjuvant chemotherapy in high-risk stage II patients [3, 4].

In contrast, the prognostic value of a tumor’s macroscopic or gross endoscopic appearance remains uncertain and remains largely underutilized in clinical practice. While previous classifications in gastric cancer linked infiltrative morphology to poor outcomes, evidence in colon cancer has been inconsistent [5]. Although some early studies reported worse outcomes in ulcerative or infiltrative morphologies [6, 7], results have been inconsistent, and the AJCC currently classifies gross morphology as a Category IV factor without established prognostic value [8].

Recent advances in high-resolution endoscopy now allow more precise assessment of tumor morphology, and some studies suggest that macroscopic features may be as prognostically important as histopathologic indicators [9]. Furthermore, studies in early-stage colorectal neoplasms, particularly laterally spreading tumors (LSTs), have demonstrated that specific morphological subtypes—such as non-granular or depressed (Paris 0-IIc) patterns—are highly predictive of deep submucosal invasion and aggressive behavior [10, 11]. These findings suggest that gross morphology reflects underlying tumor biology, a concept that may extend to more advanced, non-metastatic disease—a hypothesis that remains underexplored. For example, flat-type tumors have been shown to exhibit higher rates of microsatellite instability and poor differentiation, often correlating with worse outcomes [12]. Conversely, fungating tumors, while more amenable to detection and local treatment, demonstrate variable prognosis depending on histological features and vascular invasion status [13].

To date, few large-scale, multicenter studies have evaluated the impact of gross endoscopic morphology on long-term oncologic outcomes in non-metastatic colon cancer using contemporary data. Therefore, we conducted a large multicenter study to investigate the prognostic value of gross tumor morphology in colon cancer, focusing on its association with recurrence and survival. We hypothesized that flat/ulceroinfiltrative tumors would be associated with poorer outcomes compared to fungating/ulcerofungating tumors, and that gross morphology may serve as an independent prognostic factor to enhance existing risk stratification models.

## Methods

### Study population and data source

This multicenter retrospective cohort study was conducted using data from the colon cancer study group of the Korean Society of Gastrointestinal Cancer, comprising 9 university hospitals across South Korea. The number of cases contributed by each institution is detailed in Supplementary Table 1. All study participants were of Korean ethnicity, therefore, no further racial/ethnic distribution analysis was conducted. We initially identified patients diagnosed with and treated for colon cancer between January 2010 and December 2019.

Inclusion criteria were as follows: (1) adult patients (≥ 18 years) with histologically confirmed primary colon adenocarcinoma; (2) curative-intent endoscopic or surgical resection as the initial treatment.

Exclusion criteria were: (1) history of other cancers within 5 years prior to colon cancer diagnosis; (2) non-epithelial tumors such as lymphoma or gastrointestinal stromal tumors; or (3) incomplete medical records or loss to follow-up within 6 months of treatment, unless due to death.

### Data collection and definitions

Patient data were retrospectively extracted from electronic medical records. The following variables were collected:

1. Patient demographics: age at diagnosis, sex, body mass index (BMI), smoking and alcohol history, and family history of colorectal cancer in first-degree relatives.
2. Clinicopathological data: comorbidities (including diabetes mellitus), presenting symptoms, preoperative serum carcinoembryonic antigen (CEA) level, tumor location, and details of initial treatment (endoscopic resection, surgery alone, or surgery with adjuvant therapy). Initial treatment was classified as endoscopic resection, surgery alone, surgery with additional therapy, or chemotherapy followed by conversion surgery. The latter category includes patients who received systemic chemotherapy before surgery after multidisciplinary consultation and subsequently underwent curative-intent resection. Across participating Korean tertiary hospitals, conventional white-light colonoscopies were performed using Olympus colonoscopes (CF-H260, CF-Q180, and CF-H180/CF-HQ190 series; Olympus Corp., Tokyo, Japan) and Fujinon/Fujifilm colonoscopes (EC-450HL5, EC-450WM5, and EC-590ZW; Fujifilm Corp., Tokyo, Japan) during the study period.
3. Pathological characteristics: tumor size, histological type and grade (well, moderate, or poor differentiation), and final pathological stage based on the 7th edition of the AJCC TNM staging manual. Detailed histopathologic high-risk features, such as lymphovascular invasion, perineural invasion, and tumor budding, were not initially included in the standardized data collection protocol across the participating institutions and therefore were not available for multivariate analysis.

### Assessment of gross tumor morphology

We adopted a simplified two-category classification system designed to capture the fundamental biological distinction between tumors exhibiting predominantly luminal (exophytic) growth versus those demonstrating transmural (infiltrative) growth patterns. This binary approach was chosen because these distinct growth directions have been associated with different molecular pathways, epithelial-mesenchymal transition profiles, and clinical outcomes in prior studies [12, 14, 15]. This pragmatic classification enhances interobserver reproducibility and clinical applicability in a multicenter setting while preserving prognostic relevance.

To ensure the reliability of the assessment in this multicenter setting, endoscopic images were reviewed independently at each participating institution rather than through a central process. At each center, at least two highly experienced endoscopists, each with more than 10 years of professional experience and blinded to patient outcomes, reviewed the digitally stored high‑quality endoscopic images retrieved from the local picture archiving and communication system (PACS) obtained during the initial colonoscopy. The tumors were categorized based on their predominant macroscopic features, and in cases of discordance, the final classification was reached by consensus between the two senior reviewers.

At each participating institution, two experienced endoscopists, blinded to patient outcomes, independently reviewed high-quality endoscopic images obtained during the initial diagnostic colonoscopy and classified tumors into two categories based on their predominant macroscopic features. The tumors were categorized as follows:

1. **Flat/ulceroinfiltrative type:** tumors with a flattened appearance, central depression or ulceration, and ill-defined borders suggestive of infiltrative growth. The Flat/ulceroinfiltrative category encompasses lesions that demonstrate predominantly depressed or infiltrative growth into the bowel wall, extending the morphologic concepts of Paris classification types 0-IIc (depressed) and 0-III (excavated) into advanced stages. Diffuse infiltrative lesions, which are exceedingly rare in colon cancer, were also included in this group due to their aggressive transmural growth characteristics.
2. **Fungating/ulcerofungating type:** tumors with predominantly luminal expansion, similar to Paris type 0-I (protruded) lesions. This group primarily consists of mass-forming lesions where the dominant feature is an exophytic component rather than deep mural infiltration. To ensure a mutually exclusive classification, lesions were assigned to either group based on their most prominent endoscopic feature observed at the time of diagnosis.
3. Representative endoscopic images are presented in Supplement Fig. 1. In cases of discordance, the final classification was reached by consensus between the two reviewers.

**Fig. 1:**
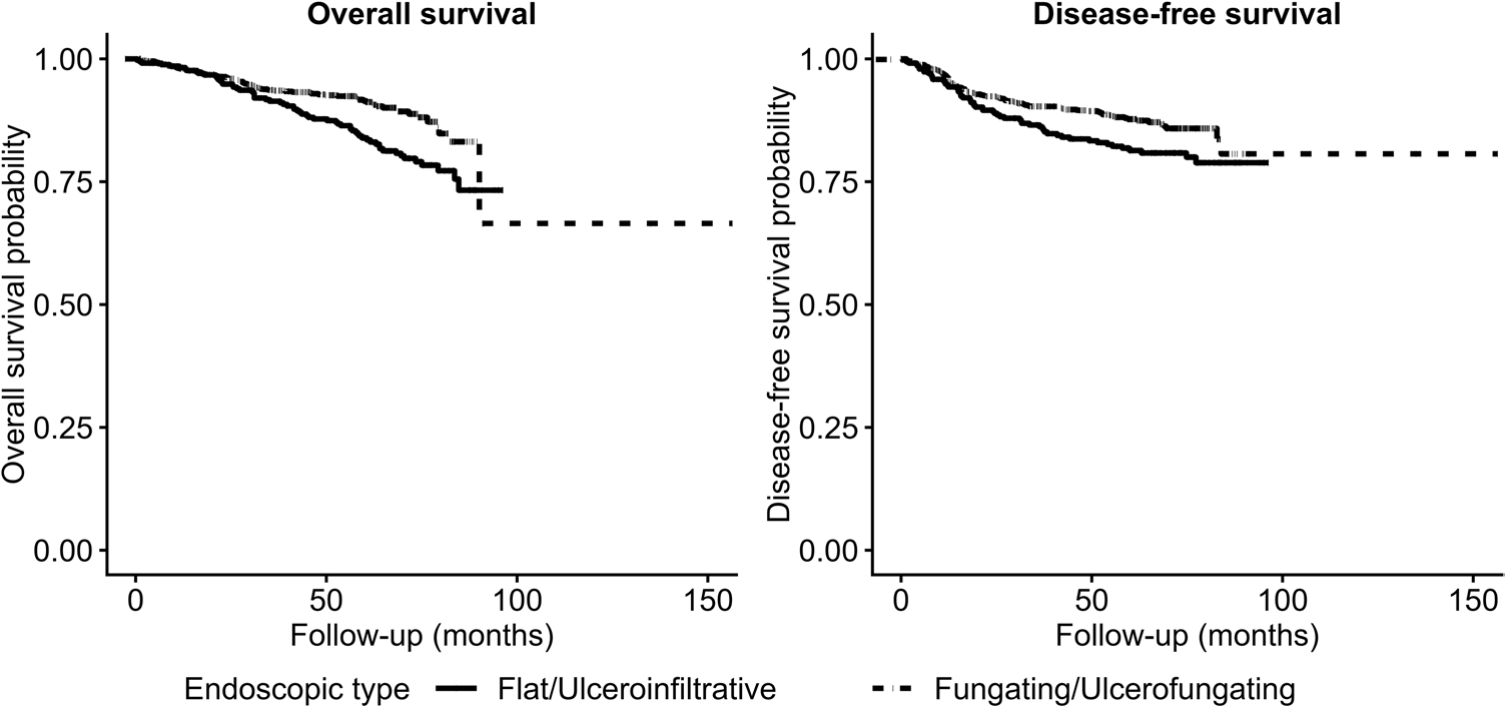
Kaplan–Meier curves for survival according to tumor morphology (**A**) Overall survival (OS)(*p* = 0.001) and (**B**) disease-free survival (DFS) (*p* = 0.024) were significantly shorter in patients with flat/ulceroinfiltrative tumors compared to those with fungating/ulcerofungating tumors. The proportional hazards assumption was assessed using Schoenfeld residuals and was satisfied for both OS (*p* = 0.71) and DFS (*p* = 0.76), confirming model validity

### Study outcomes

The primary endpoints were overall survival (OS) and disease-free survival (DFS). OS was defined as the time from curative resection to death from any cause. DFS was defined as the time from curative resection to the first documented tumor recurrence (local, regional, or distant) or death from any cause.

### Ethical considerations

This study was conducted in accordance with the principles of the Declaration of Helsinki. The study protocol was approved by the Institutional Review Board of each participating institution. Given the retrospective design and use of de-identified data, the requirement for individual informed consent was waived.

### Statistical analysis

Baseline characteristics were compared between morphological groups using the chi-square test or Fisher’s exact test for categorical variables and the Student’s t-test or Mann–Whitney U test for continuous variables. As the distribution of preoperative CEA levels was highly skewed, values for this variable are presented as median and interquartile range (IQR) rather than mean ± standard deviation to more accurately represent central tendency and variability. Survival curves for OS and DFS were generated using the Kaplan–Meier method and compared using the log-rank test. Age was categorized into two groups (< 65 years vs. ≥ 65 years) to account for the potential non-linear relationship between age and survival and to enhance clinical interpretability. This threshold of 65 years is a widely recognized cutoff in geriatric oncology, as recommended by the American Society of Clinical Oncology (ASCO) for initiating geriatric assessments, and aligns with the median age of diagnosis in our study population (63.8 years).

Independent prognostic factors were identified using a Cox proportional hazards regression model. The proportional hazards assumption of the Cox regression model was formally evaluated using Schoenfeld residual tests to ensure that hazard ratios remained constant over time. Both covariate-specific and global *p*-values were inspected, with *p* < 0.05 indicating a potential violation. Stage-specific models also satisfied the assumption (all *p* > *0.05*), confirming that hazard ratios remained constant over time and that the Cox model was appropriate for survival analyses.

The final multivariate model was adjusted for all variables listed in the table: age at diagnosis, sex, preoperative CEA level, family history of colorectal cancer, smoking history, alcohol history, hypertension, diabetes, tumor location, histologic grade, endoscopic morphology, and pathological stage. Potential collinearity between gross tumor morphology and histologic grade was assessed using generalized variance inflation factors (GVIFs) and Cramér’s V. We predefined a GVIF of < 2 and a Cramér’s V of < 0.1 as the thresholds for indicating the absence of significant multicollinearity and a negligible association between categorical variables, respectively. Analyses were performed using R software. (version 4.4.3; R Foundation for Statistical Computing, Vienna, Austria).

## Results

### Patient characteristics and tumor morphology

A total of 1,177 patients with colon cancer who underwent curative-intent endoscopic or surgical resection met the inclusion criteria and were included in the final analysis. The distribution of the cohort across the nine participating centers was relatively balanced, supporting the generalizability of our results (Supplementary Table 1). Based on endoscopic assessment, 345 patients (29.3%) were classified into the flat/ulceroinfiltrative group and 832 patients (70.6%) into the fungating/ulcerofungating group.

The baseline demographic and clinicopathological characteristics of the two groups are summarized in Table 1. No significant differences were observed between the groups in age, sex, BMI or family history of colorectal cancer in first-degree relatives. However, the flat/ulceroinfiltrative group had a significantly higher proportion of patients with a history of smoking and alcohol consumption (both *p* < 0.001). This group was also more likely to present with symptoms such as bloody stools or altered bowel habits at diagnosis (*p* < 0.001). Furthermore, preoperative carcinoembryonic antigen (CEA) levels at diagnosis were also significantly higher in this group (*p* = 0.033).

**Table 1:** Baseline characteristics

	Total (*N* = 1177)	Flat/ulceroinfiltrative (*N* = 345)	Fungating/ulcerofungating (*N* = 832)	***p***-value
Age	63.84 ± 11.39	62.06 ± 12.06	64.06 ± 11.67	0.302
Gender, male	706 (59.9%)	214 (62.0%)	492 (59.1%)	0.391
Body mass index (kg/m^2^)	23.78 ± 6.98	24.37 ± 11.87	23.53 ± 3.18	0.062
Smoking				< 0.001
never smoker	836 (71.0%)	198 (57.4%)	626 (75.2%)	
ex-smoker	153 (13.0%)	74 (21.4%)	78 (9.4%)	
current smoker	188 (16.0%)	73 (21.2%)	128 (15.4%)	
Alcohol				< 0.001
never drinker	868 (73.8%)	215 (62.3%)	653 (78.4%)	
ex-drinker	106 (9.0%)	51 (14.8%)	55 (6.6%)	
current drinker	203 (17.2%)	79 (22.9%)	124 (14.9%)	
HTN	412 (35.0%)	129 (37.4%)	283 (34.0%)	0.299
Diabetes mellitus	199 (16.9%)	57 (16.5%)	142 (17.0%)	0.887
Symptom at diagnosis				
Bowel habit change	151 (12.8%)	70 (20.3%)	81 (9.7%)	< 0.001
Bloody stool	302 (25.6%)	121 (35.1%)	181 (21.7%)	< 0.001
Family history (1st degree)	53 (4.5%)	18 (5.2%)	35 (4.2%)	0.547
CEA at diagnosis (ng/mL)	2.70 (1.39–5.60)	3.70 (1.90–8.10)	2.41 (1.23–4.90)	0.033
Location				< 0.001
Left	932 (79.1%)	307 (89.0%)	625 (75.1%)	
Pathology				0.111
Adenocarcinoma WD/MD	1140 (96.8%)	339 (98.3%)	801 (96.2%)	
Adenocarcinoma PD	37 (3.1%)	6 (1.7%)	31 (3.7%)	
T stage				< 0.001
T1	280 (23.8%)	45 (13.0%)	235 (28.2%)	
T2	229 (19.4%)	62 (18.0%)	167 (20.0%)	
T3	614 (52.1%)	223 (64.6%)	391 (46.9%)	
T4	54 (4.6%)	15 (4.3%)	39 (4.7%)	
N stage				< 0.001
N0	740 (62.9%)	158 (45.8%)	582 (70.0%)	
N1	365 (31.0%)	170 (49.3%)	195 (23.4%)	
N2	71 (6.0%)	17 (4.9%)	54 (4.6%)	
Stage				< 0.001
I	445 (37.8%)	85 (24.6%)	360 (43.2%)	
II	294 (25.0%)	73 (21.2%)	221 (26.5%)	
III	427 (36.3%)	182 (52.8%)	245 (29.4%)	
IV	11 (0.9%)	5 (1.4%)	6 (0.7%	
Type of initial treatment				< 0.001
Endoscopy	68 (5.8%)	9 (2.6%)	59 (7.1%)	
Surgery alone	599 (50.8%)	138 (40.0%)	461 (55.3%)	
Surgery with additional therapy^a^	448 (38.0%)	181 (52.5%)	267 (32.1%)	
Upfront chemotherapy followed by conversion surgery	62 (5.3%)	17 (4.9%)	45 (5.4%)	

*BMI* body mass index, *CEA* carcinoembryonic antigen, *CRC* colorectal cancer, *HTN* hypertension, *MD* moderately differentiated, *PD* poorly differentiated, *WD* well differentiated

^a^adjuvant chemotherapy or additional Radiotherapy

Pathologically, flat/ulceroinfiltrative tumors were significantly associated with more aggressive features. Compared to fungating/ulcerofungating tumors, they exhibited a more advanced T stage (*p* < 0.001), a higher incidence of lymph node metastasis (N stage, *p* < 0.001), and a more advanced overall AJCC stage (*p* < 0.001). However, there was no significant difference in the distribution of histological differentiation between the two groups (*p* = 0.111).

### Survival outcomes based on tumor morphology

The Kaplan–Meier survival analysis demonstrated significant differences in clinical outcomes between the two morphological groups (Fig. 1). Patients with flat/ulceroinfiltrative tumors had significantly shorter OS compared to those with fungating/ulcerofungating tumors (log-rank *p* = 0.001) (Fig. 1). DFS was also significantly poorer in the flat/ulceroinfiltrative group (log-rank *p* = 0.024) (Fig. 1). The proportional hazards assumption was evaluated using Schoenfeld residuals and was satisfied for both OS (*p* = 0.71) and DFS (*p* = 0.76), confirming model validity.

**Fig. 2:**
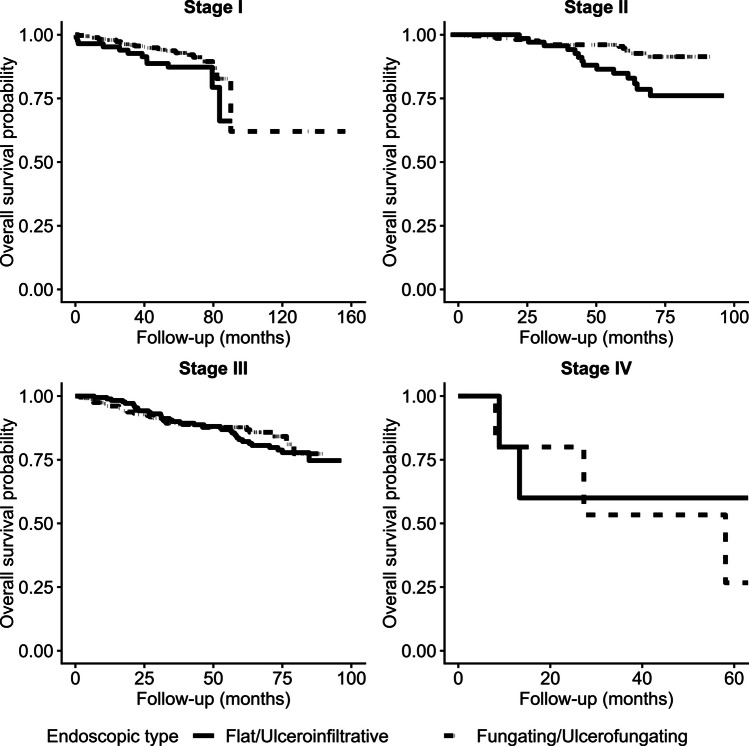
Kaplan–Meier curves for Overall Survival(OS) according to tumor morphology, stratified by pathological stage. Overall survival was compared between endoscopic types (flat/ulcerofiltrative vs. fungating/ulcerofungating) in patients with (**A**) Stage I disease (*p* = 0.130), (**B**) Stage II disease (*p* = 0.004), (**C**) Stage III disease (*p* = 0.494), and (**D**) Stage IV disease (*p* = 0.725) disease. A statistically significant difference in OS according to endoscopic morphology was observed only in patients with Stage II disease. The proportional hazards assumption was assessed using Schoenfeld residuals and no violations were observed in any stage (Stage I *p* = 0.53; Stage II *p* = 0.29; Stage III *p* = 0.12; Stage IV *p* = 0.66), confirming model validity

**Fig. 3:**
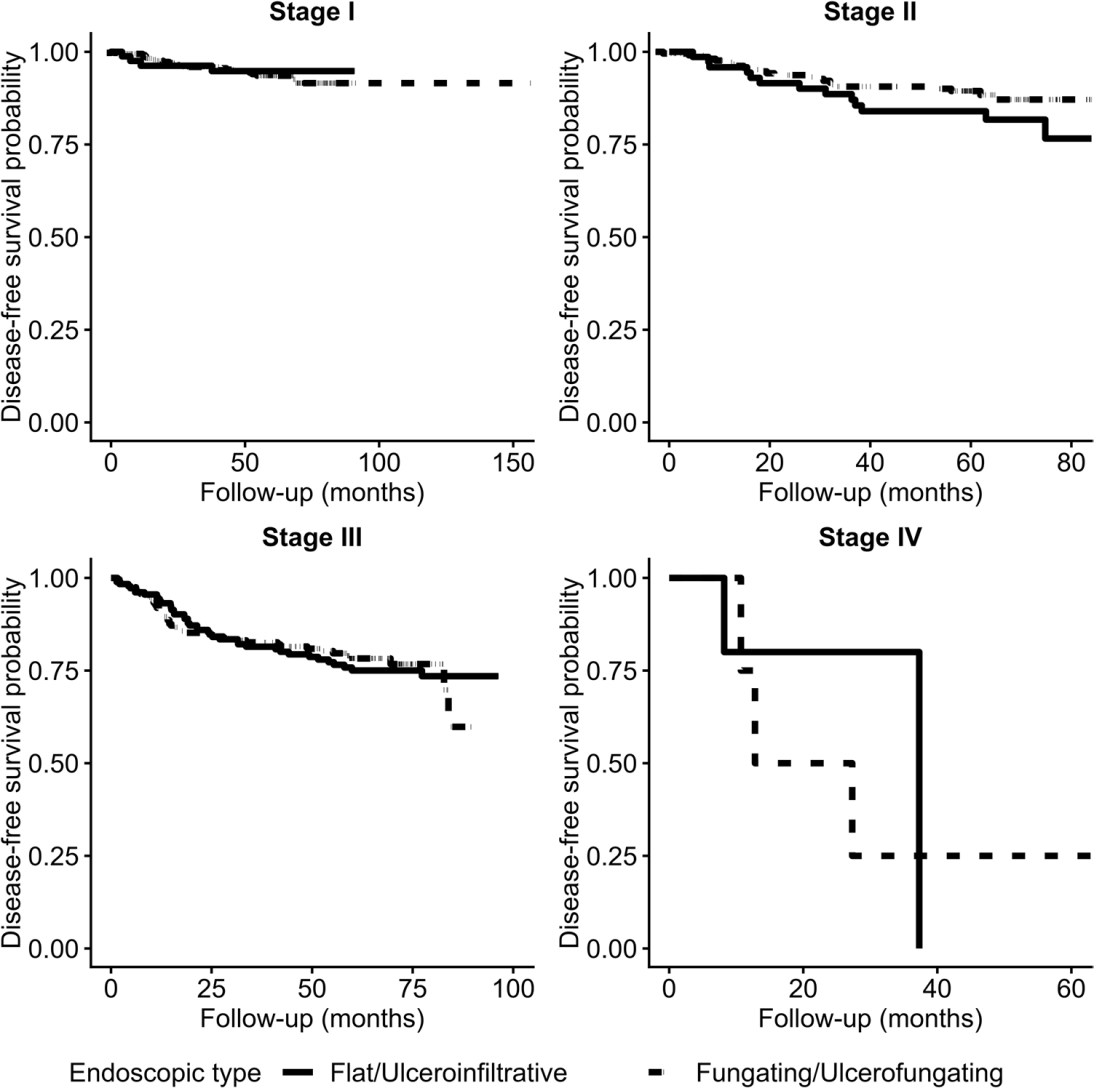
Kaplan–Meier curves for Disease-Free Survival (DFS) by endoscopic type, stratified by pathological stage. Disease-free survival was compared between endoscopic types (flat/ulcerofiltrative vs. fungating/ulcerofungating) in patients with (**A**) Stage I (*p* = 0.628), (**B**) Stage II(*p* = 0.172), (**C**) Stage III(*p* = 0.944), and (**D**) Stage IV(*p* = 0.599) disease. No statistically significant difference in DFS according to endoscopic morphology was found in any stage. The proportional hazards assumption was assessed using Schoenfeld residuals and no violations were observed in any stage (Stage I *p* = 0.07; Stage II *p* = 0.60; Stage III *p* = 0.47; Stage IV *p* = 0.99), confirming model validity

**Fig. 4:**
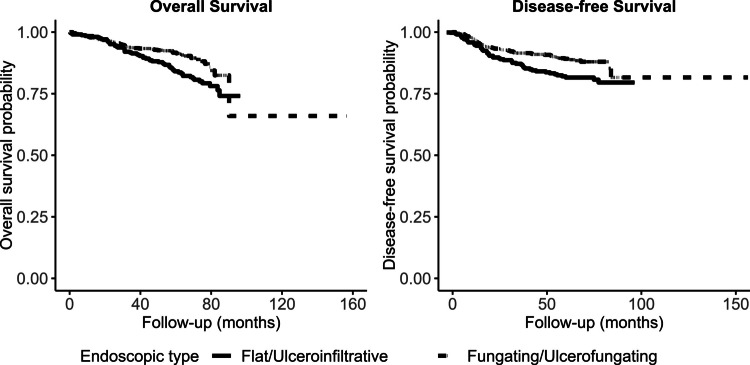
Survival outcomes according to endoscopic morphology in patients who received surgery alone or surgery with additional treatment. Kaplan–Meier curves comparing outcomes based on endoscopic type (flat/ulcerofiltrative vs. fungating/ulcerofungating) for the subgroup of patients who underwent surgery with or without additional therapy (chemotherapy or radiotherapy). In this cohort, endoscopic morphology was significantly associated with both (**A**) Overall Survival (OS, *p* = 0.005) and (**B**) Disease-Free Survival (DFS, *p* = 0.007). The proportional hazards assumption was assessed using Schoenfeld residuals and no violations were observed in either model (OS global *p* = 0.95; DFS global *p* = 0.83), confirming model validity

In a subgroup analysis stratified by disease stage, the prognostic impact of tumor morphology was most pronounced among patients with stage II colon cancer. Within this subgroup, flat/ulceroinfiltrative morphology was associated with significantly worse OS compared to fungating/ulcerofungating morphology (log-rank *p* = 0.004) (Fig. 2). In contrast, when analyzing for DFS, no statistically significant difference according to endoscopic morphology was found in any stage (Fig. 3). The proportional hazards assumption for these stage-specific Cox models was assessed using Schoenfeld residuals, and no violations were observed across all stages (all *p* > 0.05), confirming model validity for both OS and DFS. However, in a subgroup analysis of patients who received surgical treatment regardless of stage, flat/ulceroinfiltrative morphology remained a significant indicator of poorer outcomes for both OS (*p* = 0.005) and DFS (*p* = 0.007) (Fig. 4). The proportional hazards assumption was also satisfied in this subgroup (OS global *p* = 0.95; DFS global *p* = 0.83), supporting the appropriateness of the Cox model.

### Multivariate analysis of prognostic factors for survival

Multivariate Cox proportional hazards regression analysis identified four independent predictors of OS (Table 2). Gross tumor morphology remained a significant independent prognostic factor. Flat/ulceroinfiltrative morphology was associated with a 1.61-fold increased risk of death compared to fungating/ulcerofungating morphology (Hazard Ratio [HR] 1.61; 95% Confidence Interval [CI] 1.122–2.335, *p* = 0.010). Other independent predictors of poorer survival included older age (≥ 65 years, HR 1.533; 95% CI 1.066–2.206; *p* = 0.021), poor histologic differentiation (HR 5.308; 95% CI 2.901–9.711; *p* < 0.001), and more advanced pathological stage. As histologic grade and gross morphology may reflect overlapping aspects of tumor aggressiveness, potential collinearity between these variables was examined. No significant correlation was observed between the two factors (Table 1, *p* = 0.093). The GVIFs were 1.05 for histologic grade and 1.14 for gross morphology, both well below the commonly accepted threshold (GVIF < 2). The Cramér’s V between the two categorical variables was 0.055 (*p* = 0.09), indicating only a very weak association.

**Table 2:** Multivariate analysis for overall survival in all stages

	HR (95% CI)	*p*-value
Age at diagnosis (< 65 years vs. ≥ 65 years)	1.533 (1.066–2.206)	**0.021**
Sex (Female vs. Male)	1.157 (0.779–1.719)	0.470
CEA before treatment (per 1 ng/mL)	1.002 (1.000–1.004)	0.062
Family history of CRC (1st degree) (Yes vs. No)	1.498 (0.751–2.992)	0.252
Smoking_history (Never vs. Ever)	1.002 (0.640–1.567)	0.994
Alcohol_history (Never vs. Ever)	0.707 (0.457–1.094)	0.120
Hypertension (Yes vs. No)	1.250 (0.856–1.824)	0.247
Diabetes (Yes vs. No)	0.707 (0.426–1.174)	0.180
Tumor location (Right vs. Left)	0.828 (0.514–1.334)	0.438
Histologic grade (PD vs. WD/MD)	5.308 (2.901–9.711)	** < 0.001**
Gross tumor morphology (flat/ulceroinfiltratve vs. fungating/ulcerofungating)	1.619 (1.122–2.335)	**0.010**
AJCC Stage II vs I	0.897 (0.546–1.474)	**0.668**
AJCC Stage III vs I	1.347 (0.885–2.051)	**0.164**
AJCC Stage IV vs I	15.786 (6.373–39.101)	** < 0.001**

*AJCC* American joint committee on cancer, *CEA* carcinoembryonic antigen, *CRC* colorectal cancer, *CI* confidence interval, *HR* hazard ratio, *WD* well differentiated, *MD* moderately differentiated, *PD* poorly differentiated

## Discussion

In this large multicenter retrospective study, we demonstrated that the gross endoscopic morphology of colon cancer is an independent prognostic factor for overall survival (OS). It is also associated with disease-free survival (DFS) in the overall cohort, with its impact being most pronounced for OS in stage II patients. Specifically, patients with flat/ulceroinfiltrative tumors exhibited significantly worse outcomes compared to those with fungating/ulcerofungating tumors. This association remained robust after adjusting for established prognostic variables, including age, preoperative CEA levels, and final pathological TNM stage. Our findings challenge the longstanding notion that macroscopic tumor appearance lacks prognostic value in colon cancer and highlight its potential as a readily available tool for risk stratification.

The clinical implications of our results are particularly relevant within the current AJCC staging framework. Although TNM staging remains the foundation for prognostication, substantial heterogeneity exists within each stage, particularly in stage II disease, where decisions regarding adjuvant chemotherapy remain controversial. Existing high-risk features include T4 stage, lymphovascular invasion, and perineural invasion [4, 16]. Our study complements these established markers by highlighting gross tumor morphology as a previously underappreciated, yet readily identifiable, additional high-risk feature, which could be particularly valuable in refining adjuvant chemotherapy decisions for complex stage II cases.

The preoperative identification of ulceroinfiltrative morphology should prompt heightened clinical vigilance regarding the increased likelihood of locally advanced disease and aggressive tumor biology. This recognition may also support consideration of more extensive surgical strategies, including wider resection margins and more comprehensive lymphadenectomy, and improve multidisciplinary planning for personalized care. This morphological information is available immediately—well before final pathological confirmation—and can meaningfully inform early multidisciplinary treatment planning.

Our results underscore the importance of systematic endoscopic documentation of gross tumor morphology as a primary prognostic determinant, not a secondary descriptor, warranting meticulous visual characterization and systematic recording at the time of diagnosis. Given its immediate availability during initial colonoscopy, this morphological information can inform early clinical decision-making. Early recognition of flat/ulceroinfiltrative morphology may prompt heightened clinical vigilance, guiding timely discussions regarding adjuvant chemotherapy, more extensive resection, or early oncologic consultation, particularly in stage II disease with other high-risk features [9, 12, 13, 17]. For instance, cribriform or medullary morphological variants—though less common—have been linked to poor survival and may necessitate intensified treatment approaches. Recognition of these patterns at the gross level may prompt more detailed histological and molecular assessment at an early stage.

The link between infiltrative morphology and adverse outcomes likely reflects underlying molecular and cellular tumor biology. For instance, flat/ulceroinfiltrative tumors have been linked to epithelial-mesenchymal transition (EMT), which promotes loss of cell–cell adhesion and enhances insidious invasive potential [14, 18]. Furthermore, morphology may reflect distinct molecular pathways of carcinogenesis. For example, flat or non-polypoid tumors are frequently associated with microsatellite instability (MSI) and the serrated neoplasia pathway, which often occurs in right-sided colon cancers and is linked to more aggressive behavior, a finding that aligns with the well-recognized association between non-polypoid, right-sided tumors and MSI-driven serrated pathways [12, 15, 17, 19]. Similar patterns have been observed in advanced gastric cancer (Borrmann type IV), which is associated with a dismal prognosis and increased peritoneal dissemination [20]. Our findings suggest that a comparable relationship between morphology and aggressive tumor biology exists in colon cancer.

Although comprehensive molecular and pathological data—such as microsatellite instability, lymphovascular invasion (LVI), perineural invasion (PNI), and tumor budding— were not available for our entire cohort, the clinicopathologic differences observed—such as higher T and N stages in flat/ulceroinfiltrative tumors—support the hypothesis that morphology may serve as a surrogate for underlying tumor biology. Our findings align with those from studies of early-stage colorectal neoplasia, where laterally spreading tumors with non-granular or depressed morphology exhibit a higher risk of deep submucosal invasion and nodal metastasis [10, 11], demonstrating that the prognostic relevance of flat and infiltrative morphology persists beyond early lesions and continues to impact long-term outcomes following curative resection.

Our results also address inconsistencies in prior literature regarding the prognostic significance of gross morphology, which led to its current classification as a non-significant the AJCC [3]. This discrepancy is likely attributable to the limitations of earlier studies, such as their smaller sample sizes and single-center designs, in contrast to our multicenter cohort which enhances generalizability [21]. Additionally, modern high-definition endoscopy now enables more accurate and reproducible morphological classification.

From a methodological perspective, the robustness of our findings was further supported by formal model validation. The validity of the Cox proportional hazards model was confirmed through formal Schoenfeld residual testing, which demonstrated no violation of the proportional hazards assumption across all analyses. Multivariate analysis confirmed that gross morphology provides prognostic information independent of TNM staging. Moreover, diagnostic testing using generalized variance inflation factors (GVIFs) and Cramér’s V indicated no meaningful collinearity between gross morphology and histologic grade, confirming that the prognostic contribution of morphology was statistically independent of histologic differentiation.

Several limitations should be acknowledged. Firstly, the retrospective design inherently introduces selection and information biases, as the absence of randomization may have affected treatment and outcome reporting. Secondly, while morphology assessment by experienced endoscopists involved a consensus-based process at each institution, we did not provide formal measures of interobserver agreement, such as the kappa coefficient. Due to the multicenter nature of the study and the lack of a centralized image review process, providing a comprehensive concordance rate was not feasible. However, we sought to minimize subjectivity by employing a simplified binary classification and relying on the consensus of senior endoscopists with extensive clinical experience. Future studies using quantitative imaging or AI could further enhance objectivity and provide standardized reliability measures. Thirdly, we were unable to include detailed histopathologic high-risk features (e.g., lymphovascular and perineural invasion, tumor budding) were not initially included in the data collection process. The absence of these parameters, which are key for risk stratification in Stage II colon cancer, may have resulted in residual confounding and explains the limited significance observed in our DFS analysis. Fourthly, the lack of comprehensive molecular data (e.g., MSI, BRAF/KRAS mutations) for the full cohort limits our understanding of the biological mechanisms linking tumor morphology and prognosis. Fifthly, our study period (2010–2019) spanned the transition from the AJCC 7th to 8th edition staging systems. To ensure consistency, we applied the 7th edition classification. However, this does not account for tumor deposits (N1c), introduced in the 8th edition. As a result, a small number of patients classified as Stage III under the newer system may have been included in our Stage II cohort, potentially diluting prognostic differences. This suggests that the true impact of gross morphology may be even greater. Finally, the variability of adjuvant treatment protocols across institutions and over the study period is a potential unmeasured confounding factor.

In conclusion, gross endoscopic morphology is a significant and independent prognostic factor in non-metastatic colon cancer. Flat/ulceroinfiltrative morphology is strongly associated with more aggressive tumor biology and poorer survival, particularly among stage II patients. This simple, visual classification, available at initial diagnosis, has the potential to enhance risk stratification and guide personalized treatment decisions. We recommend that gross tumor morphology be systematically documented in endoscopic and pathological reports and considered for inclusion as a high-risk feature in future clinical guidelines. Prospective validation is warranted to further define its role in routine clinical practice.

## Supplementary information

Below is the link to the electronic supplementary material.

Supplementary file1 (DOCX 489 KB)

Supplementary file2 (DOCX 14 KB)

## Data Availability

The datasets generated and/or analysed during the current study are not publicly available due to patient confidentiality restrictions but are available from the corresponding author on reasonable request.
