# Supplementary material for: Impact of gross tumor morphology on the clinical outcomes of colon cancer: multicenter retrospective cohort study
Source: Int J Colorectal Dis. 2026 Feb 4;41(1):57. doi: 10.1007/s00384-026-05101-1 (PMC12872715; doi:10.1007/s00384-026-05101-1)

**Supplementary Figure 1**. Gross tumor morphology

A. Flat type


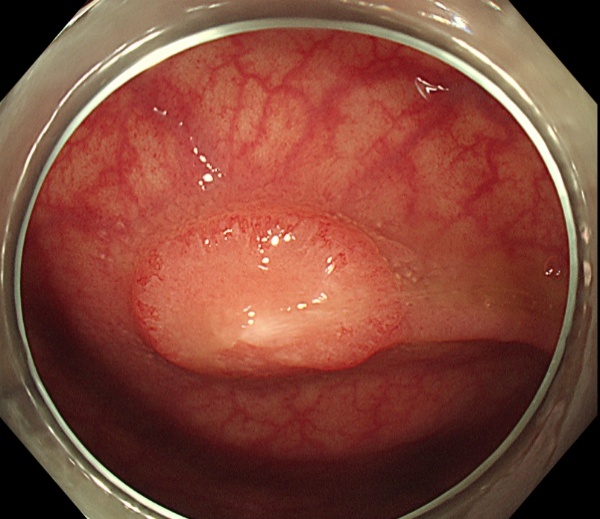


B. Ulceroinfiltrative type


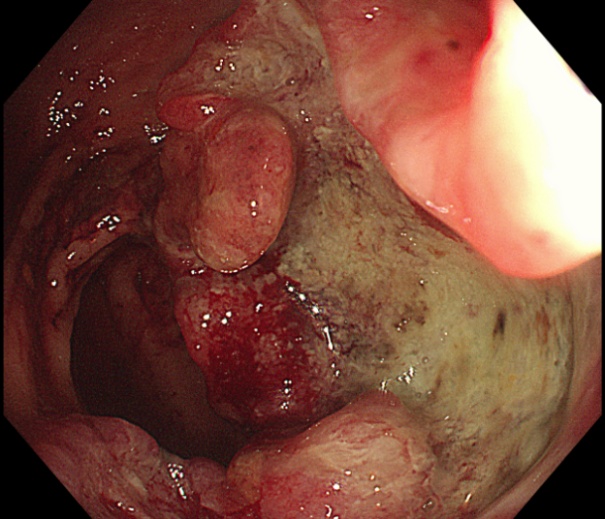


C. Fungating type


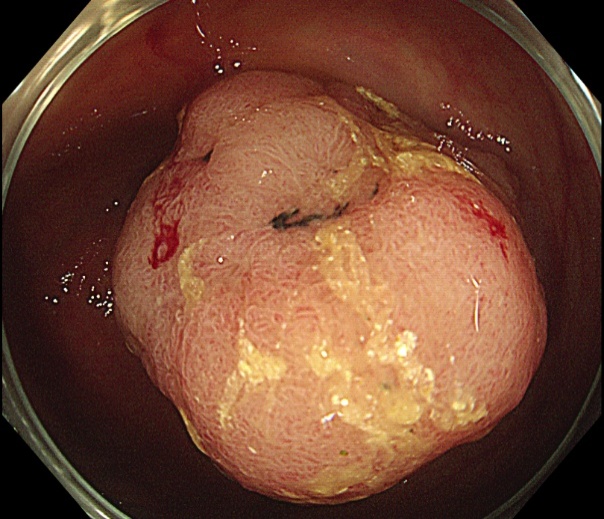


D. Ulcerofungating type


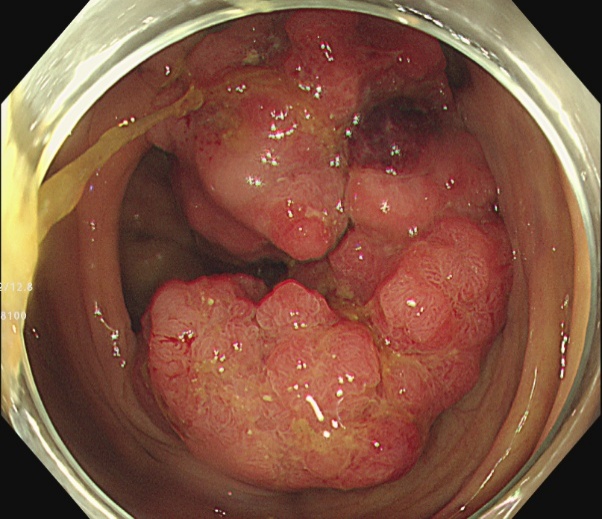

Supplement: Supplementary file 1 — Supplementary file1 (DOCX 489 KB) [file 384_2026_5101_MOESM1_ESM.docx]
