# Supplementary material for: Impact of gross tumor morphology on the clinical outcomes of colon cancer: multicenter retrospective cohort study
Source: Int J Colorectal Dis. 2026 Feb 4;41(1):57. doi: 10.1007/s00384-026-05101-1 (PMC12872715; doi:10.1007/s00384-026-05101-1)
Supplement: Supplementary file 2 — Supplementary file2 (DOCX 14 KB) [file 384_2026_5101_MOESM2_ESM.docx]

**Supplementary Table 1.** Distribution of patients by participating institution in the final analytic cohort

| **Institution (recruiting site)** | **n** |
| --- | --- |
| St. Vincent’s Hospital | 121 |
| Gangnam Severance Hospital | 88 |
| Kosin University Gospel Hospital | 192 |
| National Cancer Center | 181 |
| Soonchunhyang University Seoul Hospital | 132 |
| Yeungnam University Medical Center | 256 |
| Eulji University Hospital (Daejeon) | 65 |
| Chosun University Hospital | 49 |
| Kyungpook National University Chilgok Hospital | 93 |
| **Total** | **1177** |
